# Are primary schools ready for immersive virtual reality? Resistance among stakeholders

**DOI:** 10.1057/s41599-025-05702-1

**Published:** 2025-08-12

**Authors:** Sarah Schnyder, Josua Dubach, Lucas Dall’Olio, Sebastian Tempelmann, Trix Cacchione, Corinna S. Martarelli

**Affiliations:** 1 Faculty of Psychology, UniDistance Suisse, Brig, Switzerland; 2 University of Teacher Education Bern, Bern, Switzerland; 3 Zurich University of Teacher Education, Zurich, Switzerland; 4 University of Applied Sciences and Arts Northwestern Switzerland, Windisch, Switzerland

**Keywords:** Education, Psychology

## Abstract

Immersive virtual reality (IVR), as presented through headsets, is becoming increasingly relevant in education, especially in STEM fields, due to its potential to make complex concepts more accessible. Despite empirical evidence revealing the potential of IVR, its adoption in primary schools remains low. The objective of this paper is to examine the level of acceptance and intention to use IVR among different stakeholders in Swiss primary schools. To achieve this, we conducted online questionnaires with directors (*n* = 37), teachers (*n* = 70), and parents/caregivers (*n* = 202). The results indicated considerable variability in the responses, with a general resistance to integrating IVR being detected across all groups. Common reasons for this resistance included high costs, technical challenges, and uncertainty about IVR’s pedagogical value. However, we found that individuals who saw value in IVR were more likely to express the intention to integrate it into their schools. We discuss the importance of bridging the gap between IVR research and the reality of school implementation through targeted projects to encourage its integration into primary education.

## Introduction

Immersive virtual reality (IVR) has seen rapid advancements in recent years, with technological innovations leading to an increase in studies exploring the advantages, possibilities, and challenges of using this technology across different domains, including education. In the educational field, these studies often focus on comparing different media (e.g., headsets, desktop computers, and traditional methods) to assess learning outcomes and/or task engagement. Despite the growing body of research on the potential applications of IVR in education, there remains a significant gap between research findings and practical implementation in schools. We believe that fostering more communication and collaboration between the scientific community and stakeholders in the educational field is essential to bridge this gap and ensure that research not only informs but also supports the practical adoption in educational settings. Here, we present findings from a brief online questionnaire assessing the perception, acceptance, and integration of IVR among different stakeholders (i.e., school directors, teachers, and parents/caregivers) in Swiss primary schools. Before presenting our findings, we review the existing literature on the use and acceptance of IVR in primary school settings.

### IVR in primary school

Here, we focus on IVR displayed through headsets, which provide a wide field of view and track the user’s head movements to adjust the view accordingly. Headsets are typically paired with controllers that allow users to interact with the virtual environment. More recently, hand-tracking technology has been developed, eliminating the need for physical controllers. In general, IVR environments involve 3D graphics, spatial audio, and full-body interactions that create a sense of presence (Martarelli, Chiquet, et al., 2023). Spatial presence is an important concept in IVR research, referring to the feeling of being there in the virtual world (Witmer & Singer, 1998). Studies have shown bidirectional relationships between spatial presence and engagement with the experience (Baños et al., 2004; Chiquet et al., 2023; Riva et al., 2007; Wirth et al., 2012). Furthermore, it has been suggested that spatial presence may enhance learning via engagement (Uz-Bilgin & Thompson, 2022). The simulated experience can be comparable to that of the real world or can provide access to environments and scenarios that may be difficult or even impossible to experience in reality.

Lower costs and improved accessibility—through open-source platforms and user-friendly interfaces—have offered new opportunities for the study and implementation of IVR in education. In educational settings, both realistic simulations and imaginative scenarios have been applied across different disciplines, including the humanities, social sciences, and sciences. For instance, different IVR applications in primary schools have aimed to enhance language skills, including oral (Wu & Hung, 2022) and writing skills (Acar & Cavas, 2020). Other applications have explored cultural learning (Dall’Olio et al., 2024) through historical perspectives (López-Fernández et al., 2022) or problem-solving (Araiza-Alba et al., 2021), to name just a few. The advantage of IVR lies not only in simulating reality and allowing the user to visit different places or times but also in enabling one to have experiences that go beyond what is physically possible in the real world. This is particularly valuable for STEM disciplines, where concepts can be abstract, complex, and difficult to represent. With IVR, children can experience processes that are too small (e.g., atoms), too large (e.g., astronomical phenomena), too fast (e.g., chemical reactions), or too slow (e.g., evolution) to observe in real life. In this regard, studies have already begun to examine the impact of IVR (compared to more traditional media) on understanding scientific concepts, such as the water cycle (Liu et al., 2022; Martarelli, Dubach, et al., 2023).

Despite being less common than studies with participants of other age groups, research involving primary school children provides some evidence that IVR can significantly enhance learning outcomes. In particular, this conclusion is supported by recent meta-analyses (Lara-Alvarez et al., 2023; Villena-Taranilla et al., 2022; Yu & Xu, 2022). For example, Lara-Alvarez et al. (2023) reported a medium positive effect of virtual reality (VR, both immersive and non-immersive) on learning outcomes compared to traditional education, with a standardised mean difference of 0.64. In a similar vein, Villena-Taranilla et al. (2022) found that VR, particularly when delivered through headsets, has a notably positive impact on educational outcomes in primary schools, although the effect varies depending on the domain and duration of VR exposure. Interestingly, regarding age, they observed no significant differences, although upper primary children appeared to benefit slightly more (Villena-Taranilla et al., 2022). These findings contrast with those of other research, such as the work of Yu and Xu (2022), who found a positive impact of VR on learning outcomes, but not among primary school children. Overall, these results suggest that further research is needed on how IVR affects primary school children’s learning across different subject areas.

In addition to learning outcomes, research has also investigated the impact of IVR on task engagement (including aspects such as sense of presence, embodiment, cognitive load, motivation, self-efficacy, and self-regulation; e.g., Makransky & Petersen, 2021). For example, Laine et al. (2023) considered primary school children’s experiences using IVR as part of an environmental project and, based on a qualitative content analysis of surveys, found that children generally had positive experiences, showing enhanced physical, cognitive, and emotional engagement, and IVR could thus be perceived as influencing their learning motivation. These motivational aspects are critical because they can mediate the impact of VR (both immersive and non-immersive) on learning outcomes (Jiang & Fryer, 2023).

### Acceptance of IVR in primary school

Research shows that IVR can have positive effects on learning in primary schools, but whether it actually finds its way into the classroom depends on different factors. A key consideration is how open school directors and teachers are to IVR, as well as the attitudes of parents/caregivers toward its use. Ultimately, it is the teachers who decide whether to implement IVR in their classes, and their attitudes toward the medium play a crucial role in determining this decision (Jang et al., 2021; Nelson & Hawk, 2020). Recently, Walstra et al. (2024) analysed 11 studies from 2018 to 2023 on teachers’ perspectives on IVR integration, and their findings suggest that while teachers recognise the educational value of IVR, they have also expressed certain concerns, such as costs (Li et al., 2023), technical challenges (Patterson & Han, 2019), and time constraints (Alalwan et al., 2020). In addition to directors and teachers, parents/caregivers also play a role in shaping the adoption of technology in schools. For example, Vekiri (2010) found that children’s perceptions of parental support for technology were highly correlated with their own beliefs about information and communication technologies, including their motivation to improve their computer knowledge and skills. While parents/caregivers do not decide whether IVR will be used in primary schools, their perceived support may be related to how their children approach and experience these technologies.

To better understand the adoption of digital technologies (not specific to IVR and not specific to the classroom), several models have been developed to evaluate users’ acceptance of technology, including the Technology Acceptance Model (TAM) (Davis, 1985, 1989) and the Unified Theory of Acceptance and Use of Technology (UTAUT) (Venkatesh et al., 2003). The TAM, based on the Theory of Reasoned Action (Fishbein & Ajzen, 1975), suggests that two key factors—perceived usefulness and perceived ease of use—contribute to the intention to use a technology. Perceived usefulness refers to how much users believe the technology will enhance their work performance, while perceived ease of use is a measure of how effortless users find the technology to use. In contrast, the UTAUT contains three main predictors of the intention to use a technology: performance expectancy, effort expectancy, and social influence. Performance expectancy closely aligns with TAM’s perceived usefulness, and effort expectancy is similar to TAM’s perceived ease of use. Social influence refers to the extent to which individuals perceive that important people in their lives believe they should adopt the technology. In an early study, Davis et al. (1989) explored the relationship between the intention to use a new technology, in their case computers, and actual usage, finding that the intention to adopt the new technology predicted the self-reported actual usage. Similarly, Venkatesh et al. (2003) demonstrated that intention is a key predictor of actual usage behaviour by measuring usage through system logs to track the duration of use. Moreover, they discovered that another factor directly determining actual technology usage was facilitating conditions. Facilitating conditions refer to the available support and resources that help users in the process of technology adoption.

Although the TAM and UTAUT were developed for relatively older technologies, the UTAUT remains widely used to understand the acceptance of emerging innovations, like ChatGPT (Menon & Shilpa, 2023) and the Metaverse (Lee & Kim, 2022). Likewise, these frameworks can be used to study the adoption of IVR in schools. For instance, Lin et al. (2023) reviewed the literature on IVR acceptance among middle school directors and found that factors from the TAM significantly influenced their intention to use IVR. In a related vein, Spangenberger et al. (2023) applied the UTAUT to explore which factors influence vocational teachers’ intention to use IVR technology in the classroom. Their findings revealed that, among all the UTAUT factors, only performance expectancy significantly predicted this intention. Similarly, Boel et al. (2023) applied the UTAUT with secondary education teachers and found that all the key factors, except for effort expectancy, significantly influenced their intention to adopt IVR in the classroom. Prior to this study, Hussin et al. (2011) tested college educators and found that social influence and effort expectancy predicted their intention to use VR (both immersive and non-immersive). Collectively, these findings show that the results regarding the factors influencing the intention of teachers to use IVR are mixed. Most recently, Chiu and Zhu (2024) conducted qualitative interviews with parents/caregivers of children ranging from high school to postgraduate levels, utilising the TAM as their framework. Their findings successfully validated the original factors of the TAM. However, research on the acceptance of IVR, especially in primary schools and from different perspectives (school directors, teachers, and parents/caregivers), remains limited.

### Present study

Given the role of stakeholders in the implementation of IVR in schools, we conducted a brief online questionnaire to assess the acceptance of IVR in *primary* schools in Switzerland, focusing in particular on the perspectives of primary school directors, teachers, and parents/caregivers. Specifically, this questionnaire examined attitudes toward the use of IVR in primary school. We used the UTAUT to develop our own short questionnaire, the brevity of which was believed to ensure that we would have as many participants as possible. Additionally, for the directors and teachers, the questionnaire inquired about their intentions to implement IVR in their schools, while for parents/caregivers, it asked about their support for their child’s use of IVR in school. While previous research has primarily focused on identifying factors influencing the intention to use IVR (e.g., Lin et al., 2023; Spangenberger et al., 2023), our study aimed to assess overall acceptance. Since we expected varying sample sizes among stakeholder groups, our goal was not to compare them directly but rather to evaluate the general level of acceptance of IVR in primary education. Given the gap between research on the use of IVR in education and its actual implementation—currently nonexistent in primary schools in Switzerland—we hypothesised a relative resistance to the use of IVR in primary schools. We were also interested in exploring the associations between attitudes toward the use of IVR in primary schools and the intentions to use IVR or support for its implementation, as well as identifying the most common reasons cited for low intentions or actual support.

## Methods

### Transparency and openness

In this section, we report on how we determined our sample size, as well as all the data exclusions, manipulations, and measures applied in the study. The data, code, and research materials are available at the Open Science Framework and can be accessed at https://osf.io/jpq9n/. The study was not preregistered.

### Participants

A total of 309 individuals participated in the study without receiving any incentives. Participant demographics are reported in Table 1. All participants resided in Switzerland, with the majority being German speakers (93.53%). Additional information about the samples is provided in the Supplementary Material.

**Table 1:** Participant demographics and IVR experience.

Stakeholder	*N* (% of total)	Female participants *N* (% of sample)	Mean Age (SD)	Never used IVR (%)
Directors	37 (11.97%)	18 (48.65%)	47.76 (SD = 13.18)	67.57%
Teachers	70 (22.65%)	48 (68.57%)	40.11 (SD = 14.66)	81.43%
Parents/caregivers	202 (65.37%)	136 (67.33%)	44.06 (SD = 11.37)	66.34%

### Measures

The main items of the questionnaire were the same for directors, teachers, and parents/caregivers. Specifically, they were asked to indicate their level of agreement with several statements related to the integration of IVR into primary education. An overview of the items presented to the different stakeholder groups is provided in Table 2. Please note that while we use the term IVR in this paper, we used VR headsets in the questionnaires to facilitate participants’ understanding. VR was defined as a computer-generated environment that users can immerse themselves in using special headsets and, in some cases, additional accessories such as controllers. Thus, in the questionnaire items, VR referred specifically to IVR, as per our definition.

**Table 2:** Items of the main questionnaire.

Item in questionnaire	Contained in questionnaire for	Format of answer	Adapted from
Virtual reality (VR) applications should be integrated into primary school teaching.	Directors, teachers, parents/caregivers	Agreement from 0 to 100	-
I am confident that VR can be practically applied in primary school.	Directors, teachers	Agreement from 0 to 100	-
VR has great potential to improve learning in primary school.	Directors, teachers, parents/caregivers	Agreement from 0 to 100	Performance expectancy in UTAUT (Venkatesh et al., 2003)
I have the necessary resources to be able to use VR at my school.	Directors, teachers	Agreement from 0 to 100	Facilitating conditions in UTAUT (Venkatesh et al., 2003)
I have the necessary knowledge to be able to use VR at my school.	Directors, teachers	Agreement from 0 to 100	Facilitating conditions in UTAUT (Venkatesh et al., 2003)
People whose opinions are important to me support the use of VR at my school.	Directors, teachers	Agreement from 0 to 100	Social influence in UTAUT (Venkatesh et al., 2003)
I think it is easy to use VR in teaching.	Directors, teachers	Agreement from 0 to 100	Effort expectancy in UTAUT (Venkatesh et al., 2003)
In order to use VR applications in teaching, teachers need further training/introduction.	Directors, teachers	Agreement from 0 to 100	Effort expectancy in UTAUT (Venkatesh et al., 2003)
The use of VR in teaching carries risks.	Directors, teachers, parents/caregivers	Agreement from 0 to 100	-
How likely is it that you will use VR applications in your school in the future?	Directors, teachers	Probability from 0% to 100%	Behavioural intention in UTAUT (Venkatesh et al., 2003)
How strongly do you support the use of VR in primary school classes for your child/children?	Parents/caregivers	Support from 0 to 100	-

The questionnaire addressed several aspects that were aligned with the UTAUT. Directors and teachers were asked about their perceptions of IVR’s potential to enhance learning, which reflected the *performance expectancy* construct (item: “VR has great potential to improve learning in primary school”). They were then tasked with evaluating whether they had the necessary resources and knowledge to implement IVR in their schools, which corresponded to the *facilitating conditions* (items: “I have the necessary resources to be able to use VR at my school” and “I have the necessary knowledge to be able to use VR at my school”). The questionnaire also examined *social influence* by asking the directors and teachers whether individuals whose opinions were important to them supported their use of IVR in education (item: “People whose opinions are important to me support the use of VR at my school”). Additionally, the directors and teachers assessed how easy it would be to integrate IVR into teaching at their respective schools and the necessity for further training of teachers for the effective use of the technology, which pertained to *effort expectancy* (item: “I think it is easy to use VR in teaching” and “In order to use VR applications in teaching, teachers need further training/introduction”). In addition to these constructs from the UTAUT, the questionnaire included questions on the general appropriateness of integrating IVR into primary school lessons (item: “VR applications should be integrated into primary school teaching”), their confidence in the practical application of IVR (item: “I am confident that VR can be practically applied in primary school”) and their views on the potential risks associated with using IVR in the classroom (item: “The use of VR in teaching carries risks”). For all these items, a visual analogue scale ranging from 0 to 100 was used, where 0 represented “I do not agree at all,” 50 signified “I neither agree nor disagree,” and 100 indicated “I fully agree.”

Furthermore, directors and teachers were asked to estimate the likelihood of using IVR applications in their respective schools/classrooms in the future, on a scale from 0% (“I will definitely not use VR in my school/classroom in the future”) to 100% (“I will definitely use VR in my school/classroom in the future”). This question measured *behavioural intention* from the UTAUT. If they indicated that they were unlikely to use IVR, they were required to select the main reasons from a list, which included options such as “lack of time,” “technical challenges,” “high costs,” “concerns about student distraction,” “uncertainty regarding pedagogical/didactic advantages,” “additional work for the teacher,” “incompatibility with the curriculum,” “too little control over what students do in VR,” and “other.” For those selecting “other,” a text field was provided to articulate additional reasons.

The questionnaire for parents/caregivers was designed to be as similar as possible to the one used for directors and teachers. Parents/caregivers were asked the same questions about their perceptions of IVR’s potential to enhance learning (*performance expectancy*), the general appropriateness of integrating IVR into primary school lessons, and their views on the potential risks associated with using IVR in the classroom. All other items from the directors’ and teachers’ questionnaires were not applicable to parents/caregivers. In addition, parents/caregivers were asked how strongly they supported the use of IVR in primary school classes for their child/children. They rated their level of support from 0 to 100, where 0 meant “I do not support the use of VR in primary school classes for my child/children at all,” and 100 meant “I do support the use of VR in primary school classes for my child/children very much.” For parents/caregivers who were opposed to their child/children using IVR in the classroom, a follow-up question was included to identify their primary reasons. The list of reasons was identical to the one presented to directors and teachers who were unlikely to use IVR in their schools.

### Procedure

We aimed to reach as many participants as possible between February and September 2024 through personal contacts in the French- and German-speaking parts of Switzerland. The study employed a convenience sampling approach, meaning that participants were not randomly selected but rather recruited based on accessibility and willingness to participate. To be eligible for the study, participants had to be primary school directors, teachers, or parents/caregivers of Swiss primary school children (children aged between 6 and 12 years). Participants were excluded if they did not meet the eligibility criteria or if they did not complete the questionnaire in full. Out of 468 participants who began the questionnaire, 309 completed it, resulting in a completion rate of 67.47%. Specifically, 65 directors started the questionnaire, with 37 (56.92%) completing it; 116 teachers started it, with 70 (60.34%) completing it, and 277 parents/caregivers started it, with 202 (72.92%) completing it.

The online questionnaires for the directors, teachers, and parents/caregivers were programmed using the open-source software LimeSurvey (https://www.limesurvey.org) and took an average of five minutes to complete. After a short explanation of what IVR is, the stakeholders were prompted to answer the main questionnaire items, followed by general questions about their age, gender, frequency of IVR use, and quality of experience with IVR. Directors were asked about the number of classes, teachers, and students at their schools, as well as their years of experience in school management. Teachers were asked about their media use both in the classroom and privately, the subjects and grades they taught, and their years of teaching experience. Parents/caregivers were asked about their child’s age, their child’s media use, and their own media usage.

### Analytical approach

Data were analysed using R, version 4.3.2 (R Core Team, 2023). We conducted a descriptive analysis of the main items, separated by the different stakeholders. Responses regarding attitudes toward IVR were visualised categorically to identify response tendencies across multiple items from directors, teachers, and parents/caregivers. For intention/support to use IVR, raincloud plots were employed to illustrate the distribution and variability of the responses. Our approach was primarily descriptive. In addition, we carried out one-sample t-tests comparing responses to a chance level of 50%. Two-tailed p-values were used for all tests. Given the large number of tests, our aim was to identify a general pattern (i.e., whether resistance existed) rather than to focus on specific comparisons between the different dimensions measured by the items. The inferential statistics aligned with the descriptive statistics; thus, we report these results in the Supplementary Material.

In an explorative manner, we also descriptively analysed the reasons provided by the stakeholders (directors, teachers, and parents/caregivers) regarding why they believed IVR headsets might not be feasible for implementation in primary school settings. Finally, we computed Pearson’s correlations between the variables of interest, specifically between the acceptance of IVR and the intention to use IVR in primary schools (directors and teachers), and support for using IVR in primary schools (parents/caregivers).

## Results

### Acceptance of IVR in primary school

**Fig. 1:**
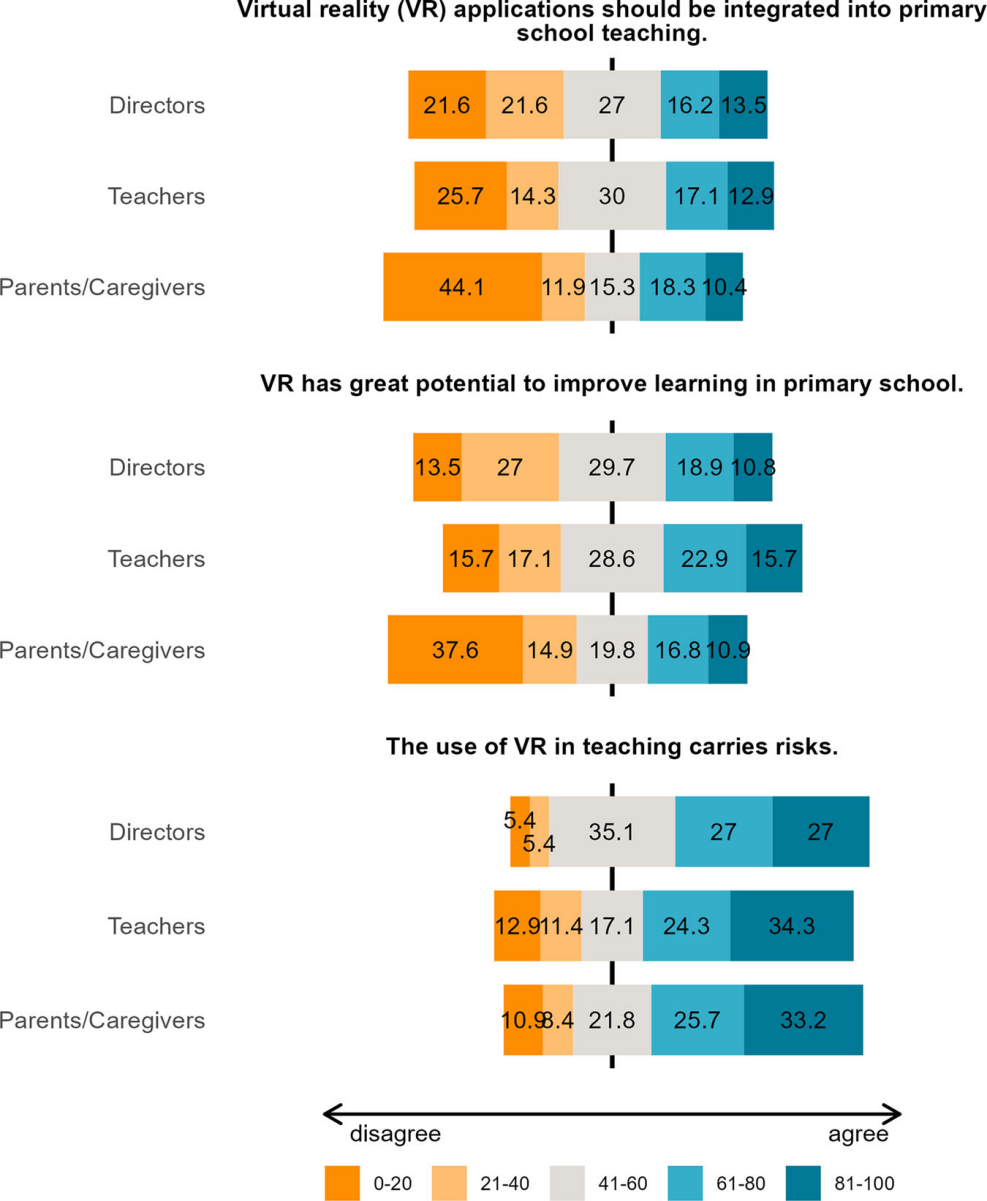
Ratings of directors (*n* = 37), teachers (*n* = 70), and parents/caregivers (*n* = 202) for different items. The numbers represent the percentages of negative (orange), rather negative (light orange), neutral (grey), rather positive (light blue), and positive (blue) answers. These variables were measured using analogue Likert scales, with 0 indicating full disagreement and 100 indicating full agreement with the respective items. Note that while the questionnaire used the term “VR”, it was explicitly defined as IVR (VR presented through headsets) to ensure participant understanding.

**Fig. 2:**
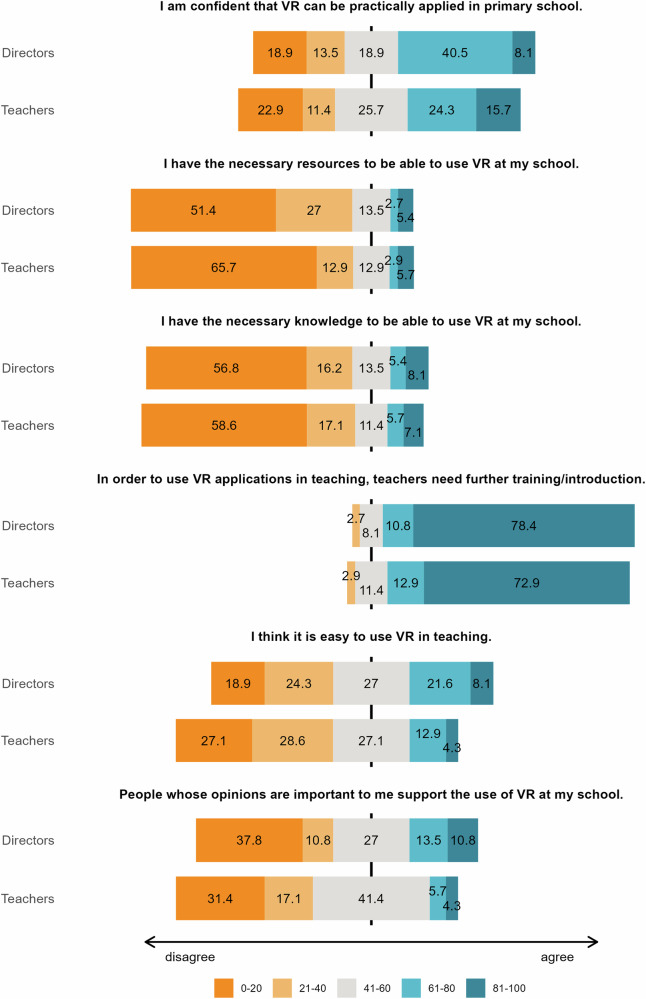
Ratings of directors (*n* = 37) and teachers (*n* = 70) for different items. The numbers represent the percentages of negative (orange), rather negative (light orange), neutral (grey), rather positive (light blue), and positive (blue) answers. These variables were measured using analogue Likert scales, with 0 indicating full disagreement and 100 indicating full agreement with the respective items. Note that while the questionnaire used the term “VR”, it was explicitly defined as IVR (VR presented through headsets) to ensure participant understanding.

Overall, the results indicated a general tendency toward resistance, although responses ranged from resistance to acceptance across the different items assessed in our study. We report the descriptive statistics in Figs. 1 and 2, and the one-sample t-tests in the Supplementary Material. Regarding the integration of IVR applications into primary schools, the directors’ responses showed a relatively balanced distribution. Specifically, 27.0% of directors were neutral, 29.7% agreed to some extent (16.2% somewhat agreed, and 13.5% strongly agreed), and a slight majority of 43.2% disagreed (21.6% somewhat disagreed, and 21.6% strongly disagreed). A similar trend was observed among teachers, with 30.0% neutral, 30.0% agreeing (17.1% somewhat agreed, and 12.9% strongly agreed), and 40.0% disagreeing (14.3% somewhat disagreed, and 25.7% disagreed). In contrast, a more polarised response was found among parents/caregivers, with 15.3% being neutral, 56.0% disagreeing (11.9% somewhat disagreed, and 44.1% strongly disagreed), and only 28.7% expressing agreement (18.3% somewhat agreed, and 10.4% strongly agreed).

When it came to the potential of IVR to improve learning in primary school, a slight majority of directors viewed IVR unfavourably, with 40.5% disagreeing (27.0% somewhat disagreed, and 13.5% strongly disagreed) and 29.7% agreeing (18.9% somewhat agreed, and 10.8% strongly agreed). Teachers were more optimistic, with 38.6% agreeing (22.9% somewhat agreed, and 15.7% strongly agreed) and 32.8% in disagreement (17.1% somewhat disagreed, and 15.7% strongly disagreed). Among parents/caregivers, a mix of opinions was observed, but overall, they were more sceptical, with 52.5% disagreeing (14.9% somewhat disagreed, and 37.6% strongly disagreed) and 27.7% agreeing (16.8% somewhat agreed, and 10.9% strongly agreed).

For the statement, “The use of VR in teaching carries risks,” a clear majority of directors agreed, with 54.0% in agreement (27.0% somewhat agreed, and 27.0% strongly agreed), while only 10.8% disagreed (5.4% somewhat disagreed, and 5.4% strongly disagreed). Teachers also shared this concern, with 58.6% agreeing (24.3% somewhat agreed, and 34.3% strongly agreed), and 24.3% disagreeing (12.9% somewhat disagreed, and 11.4% strongly disagreed). A similar trend was seen among parents/caregivers, with 58.9% agreeing (25.7% somewhat agreed, and 33.2% strongly agreed) and 19.3% disagreeing (8.4% somewhat disagreed, and 10.9% strongly disagreed).

The other questions were only asked of directors and teachers (see Fig. 2). Both groups, despite expressing confidence that IVR could be used in primary schools (48.6% of directors, with 40.5% somewhat agreeing, and 8.1% strongly agreeing, and 40.0% of teachers, with 24.3% somewhat agreeing, and 15.7% strongly agreeing), evaluated that they did not have the necessary resources (78.4% of directors, with 27.0% somewhat agreeing, and 51.4% strongly agreeing, and 78.6% of teachers, with 12.9% somewhat agreeing, and 65.7% strongly agreeing) or knowledge (73.0% of directors, with 16.2% somewhat agreeing, and 56.8% strongly agreeing, and 75.7% of teachers, with 17.1% somewhat agreeing, and 58.6% strongly agreeing) to implement IVR in their schools. Reflecting these responses, both directors and teachers considered further training essential for the use of IVR in primary schools (89.2% for directors, with 10.8% somewhat agreeing, and 78.4% strongly agreeing, and 85.8% for teachers, with 12.9% somewhat agreeing, and 72.9% strongly agreeing). For the statement, “I think it is easy to use VR in teaching,” responses were somewhat less skewed, with 29.7% of directors agreeing (21.6% somewhat agreed, and 8.1% strongly agreed) and 17.2% of teachers agreeing (12.9% somewhat agreed, and 4.3% strongly agreed). Regarding the final statement related to social influence (i.e., asking directors and teachers whether individuals whose opinions were important to them support their use of IVR in education), the pattern was similar: 24.3% of directors agreed (13.5% somewhat agreed, and 10.8% strongly agreed), compared to 10.0% of teachers (5.7% somewhat agreed, and 4.3% strongly agreed).

### Intention/support for using IVR in primary schools in the future

**Fig. 3:**
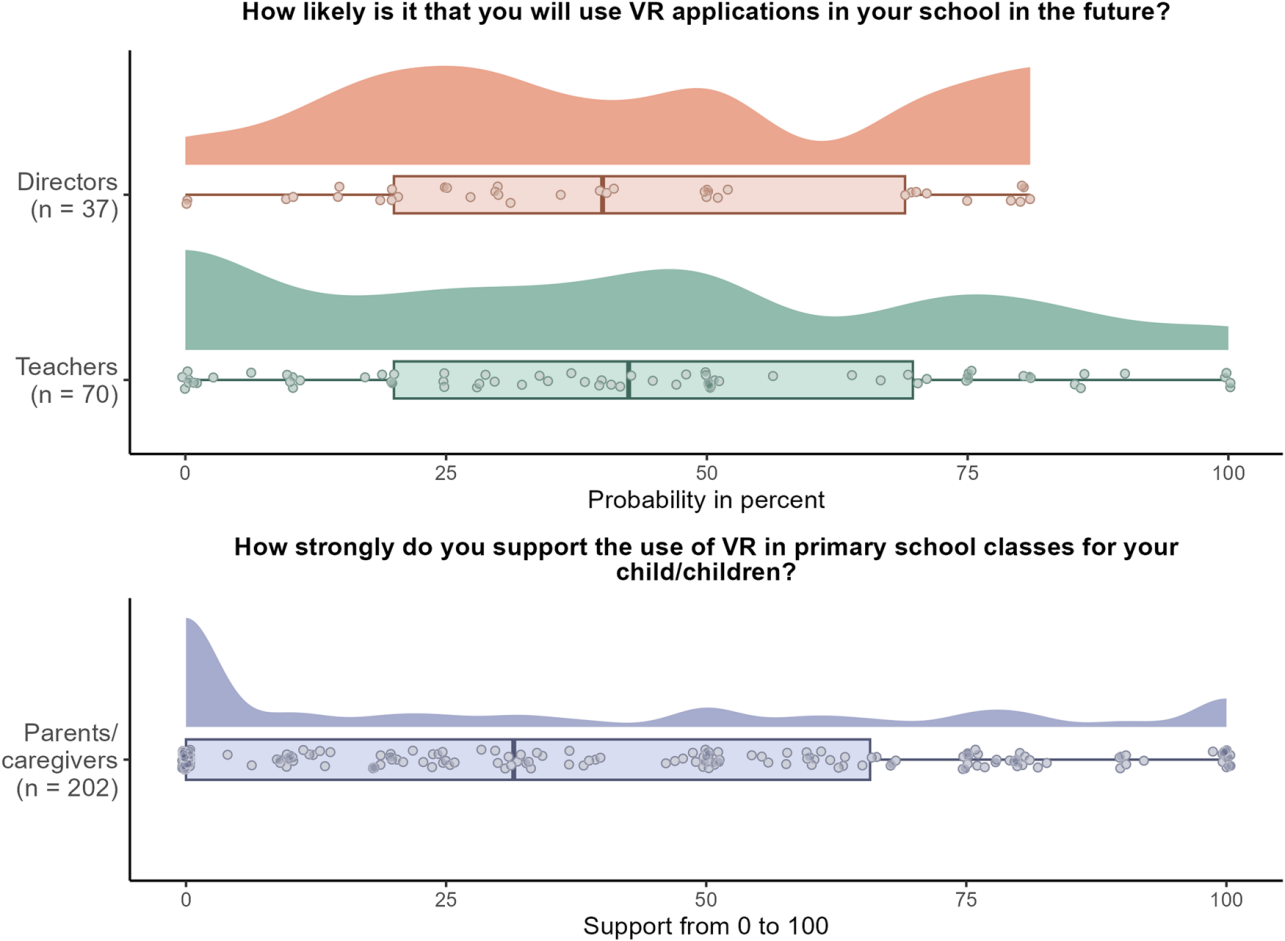
Intention/support for the use of IVR in primary schools among directors, teachers, and parents/caregivers. Raincloud plots in the top panel illustrate the likelihood that the directors (*n* = 37) and teachers (*n* = 70) would use IVR applications in their schools in the future, with probability ratings ranging from 0 to 100. The raincloud plot in the bottom panel shows the support that parents/caregivers (*n* = 202) expressed for using IVR in primary school classes for their child/children. For each stakeholder, the plot shows the cloud of dots (individual data points), boxplots (thick lines, with the vertical line representing the median value), and half-violin plots (showing the density distributions). Note that while the questionnaire used the term “VR”, it was explicitly defined as IVR (VR presented through headsets) to ensure participant understanding.

The data for this aspect are displayed in Fig. 3. One-sample *t*-tests were performed, the results of which can be found in Appendix B. Directors (mean score of 41.68 out of 100, median of 40.00) and teachers (mean score of 44.04 out of 100, median of 42.50) were generally not likely to adopt IVR applications in primary schools in the future. Moreover, parents/caregivers (mean score of 37.58 out of 100, median of 31.50) did not support its use. However, as also shown in Fig. 3, the range of responses was broad, with directors reporting scores from 0 to 81, and both teachers and parents/caregivers covering the full possible range.

### Reasons against using/supporting IVR in primary schools

**Fig. 4:**
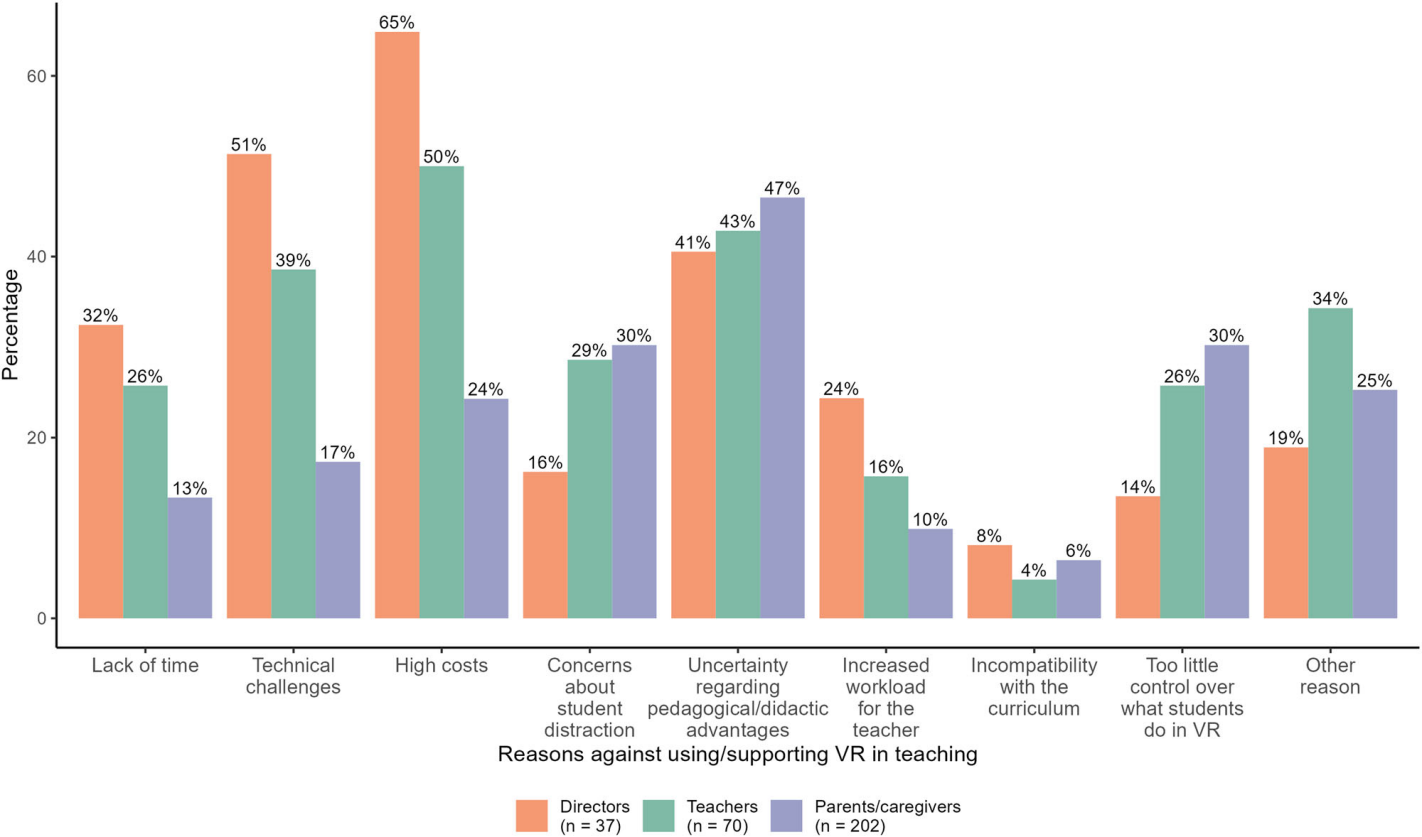
Frequency of reasons against the use/support of IVR in primary schools. This figure illustrates the selection frequency (in percentages) for various reasons opposing the use and support of IVR in primary schools, categorised by stakeholders: directors (orange), teachers (green), and parents/caregivers (blue).

The frequencies of the reasons selected by directors, teachers, and parents/caregivers for not adopting or supporting IVR in primary schools are displayed in Fig. 4. Among the directors, the most frequently mentioned reason was the high cost associated with implementing IVR, with 64.86% of directors selecting this as a barrier. This was closely followed by concerns about technical challenges (51.35%). Other notable factors were uncertainty regarding pedagogical/didactic advantages (40.54%), concerns regarding lack of time for implementation (32.43%), and the potential for increased teacher workload (24.32%). All other reasons were cited by fewer than 20% of the directors.

The teachers shared many of these concerns, with high costs ranking as their primary issue, as reported by 50.00% of the respondents. Additionally, uncertainty regarding pedagogical/didactic advantages (42.86%) and technical challenges (38.57%) were frequently cited reasons. Teachers also expressed concerns about the potential for student distraction (28.57%), too little control over what students do in IVR (25.71%), and lack of time (25.71%). All other reasons were selected by less than 20% of the teachers.

For parents/caregivers, the leading reason for the low support for IVR in primary schools was uncertainty regarding its pedagogical and didactic advantages (46.53%). This was followed by concerns about student distraction (30.20%) and too little control over what students do in IVR (30.20%). Additionally, high costs were a concern for 24.26% of parents/caregivers. Again, fewer than 20% of parents/caregivers cited all other reasons.

### Associations between acceptance of IVR and intention to use IVR in primary schools

**Fig. 5:**
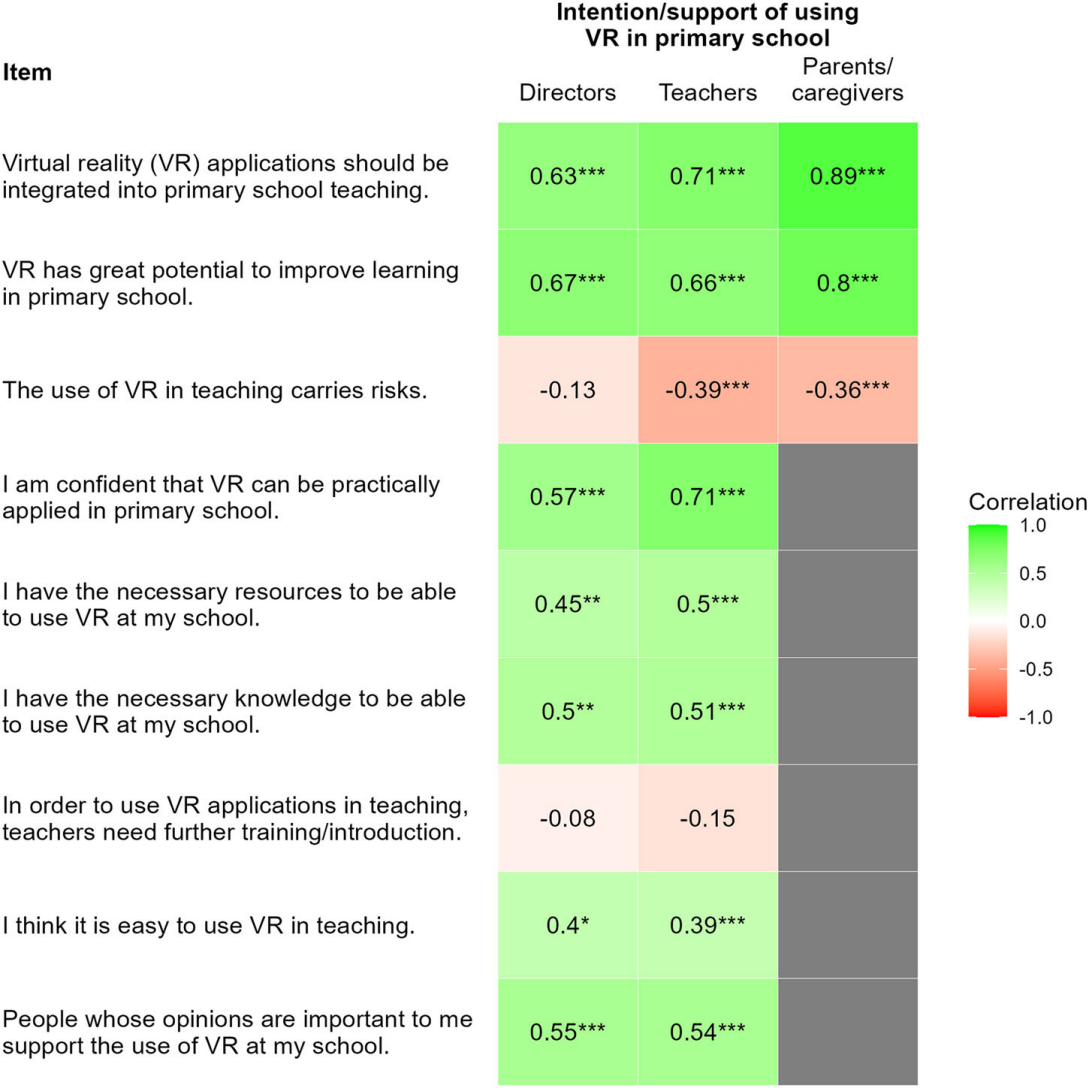
Pearson’s correlation matrix between different items and (1) the intention to use IVR in primary schools by directors (*n* = 37) and teachers (*n* = 70), and (2) the support for using IVR in primary schools by parents/caregivers (*n* = 202), respectively. Note that while the questionnaire used the term “VR”, it was explicitly defined as IVR (VR presented through headsets) to ensure participant understanding. Green cells represent positive correlations, and red cells represent negative correlations. *p < 0.05. **p < 0.01. ***p < 0.001.

The correlations in Fig. 5 reveal that certain associations emerged in the expected directions. Specifically, there were positive associations between the acceptance of IVR and the intention to use IVR across all groups, including directors, teachers, and parents/caregivers. For instance, the correlation between the belief that “VR applications should be integrated into primary school teaching” and the intention to use IVR was particularly strong, with *r*(35) = 0.63, *p* < 0.001 for directors, *r*(68) = 0.71, *p* < 0.001 for teachers, and *r*(200) = 0.89, *p* < 0.001 for parents/caregivers. Similarly, the belief in IVR’s potential to improve learning also showed high correlations with the intention to use IVR, such as *r*(35) = 0.67, *p* < 0.001 for directors, *r*(68) = 0.66, *p* < 0.001 for teachers, and *r*(200) = 0.80, *p* < 0.001 for parents/caregivers. Conversely, there were negative associations between concerns about the risks of IVR and the intention to use it. For example, the perception that “the use of VR in teaching carries risks” correlated negatively with the intention to use IVR, particularly for teachers, *r*(68) = −0.39, *p* < 0.001 and parents/caregivers, *r*(200) = −0.36, *p* < 0.001 indicating that greater concern about risks may reduce support for IVR adoption. Additionally, there were small negative correlations regarding the need for additional teacher training in using IVR, with *r*(68) = −0.15, *p* = 0.214 for teachers, and *r*(35) = −0.08, *p* = 0.654 for directors, suggesting that while some saw the need for additional training, this itself did not correlate with their overall support for IVR. Generally, these correlations indicate that the more likely stakeholders were to view IVR as beneficial and feasible to implement, the stronger their intentions to use it, whereas concerns about risks were associated with reduced intentions, but to a lesser extent.

## Discussion

In this study, we investigated the attitudes of directors, teachers, and parents/caregivers toward the use of IVR in primary schools. The results revealed mixed attitudes, with both directors and teachers showing a broad range of opinions and a significant portion expressing resistance to IVR integration, although some were open to its potential benefits. Parents/caregivers were more sceptical overall, particularly regarding the risks and disadvantages of IVR in education. Consistent with previous studies (e.g., Alalwan et al., 2020; Li et al., 2023; Patterson & Han, 2019), costs, technical challenges, and uncertainty about IVR’s educational value were the most frequently cited barriers to IVR’s adoption among directors and teachers. High costs were also a concern for parents/caregivers, although they were less focused on the financial aspect and more on the potential distractions and control over children’s activities in IVR environments. Furthermore, the directors and teachers largely believed that they lacked the necessary resources and knowledge to implement IVR effectively, which highlighted the need for further training in areas such as understanding how to integrate IVR into the curricula and technical skills. Regarding future adoption, the likelihood of integrating IVR into primary schools was low, with school directors, teachers, and parents/caregivers generally not supporting its use.

To understand those factors that might influence IVR adoption, referring to established theories, such as the UTAUT, can be helpful (Venkatesh et al., 2003). Accordingly, our study included several items influenced by the UTAUT to capture those factors determining the intention to use a technology and its actual usage, which revealed patterns consistent with prior research on IVR acceptance in schools. Notably, we found a positive correlation between performance expectancy (perceptions of IVR’s potential to enhance learning) and the intention to adopt IVR, which aligned with findings reported by Spangenberger et al. (2023) and Boel et al. (2023). Additionally, Boel et al. (2023) highlighted social influence (perceived support from key individuals) and facilitating conditions (availability of resources and knowledge) as significant predictors of IVR adoption intention. Hussin et al. (2011) also found that effort expectancy (the belief that IVR is easy to implement) positively predicted the intention to use IVR in education. Our findings echo these results, with positive correlations being detected between these aspects and the intention to implement IVR in primary schools. Beyond the UTAUT framework, our findings show that positive attitudes toward the appropriateness of IVR in primary education and confidence in its practical application were also positively correlated with the intention to use IVR. Conversely, concerns about potential risks were negatively correlated with this intention.

A key finding of our study is the relative resistance to the use of IVR in primary school among directors, teachers, and parents/caregivers. Given the limited research examining the acceptance of IVR in primary schools, it is challenging to compare our findings with those of existing studies, as most research emphasises the factors influencing acceptance rather than measuring the level of acceptance itself. However, parallels can be drawn with the review by Walstra et al. (2024), which highlights both hesitance and enthusiasm among primary school teachers regarding IVR integration. This duality is reflected in our results, where, despite the general resistance, responses to most items showed considerable variability. The relative scepticism in our study is likely rooted in the limited use of IVR technology in educational settings and other domains. Previous research has suggested that experience with a technology plays a significant role in shaping individuals’ perceptions and use of it (Khlaif, 2018; Taylor & Todd, 1995; Wilfong, 2006). For instance, Li et al. (2023) demonstrated that the intention to incorporate IVR into educational settings improved significantly after about 15 minutes of engagement with an IVR education programme. The study suggests that, without firsthand experience, stakeholders might remain cautious or resistant to its adoption. These findings imply that a gradual integration of IVR into primary school education could help address the current resistance. Interestingly, this relative resistance contrasts with the experiences of children, as research suggests that when children are given the opportunity to use IVR headsets, they generally respond positively (e.g., Martarelli, Dubach, et al., 2023; Sun et al., 2017). Specifically, it has been shown that primary school children enjoy working with this technology. Nevertheless, high costs and technical challenges can still be strong reasons for relative resistance. However, these barriers are not impossible to overcome; reducing costs and providing teachers with adequate support and training could facilitate the integration of IVR into primary education.

Although evidence regarding the direct impact of IVR on learning outcomes is mixed, some studies indicate that IVR can enhance learning (see, e.g., Lara-Alvarez et al., 2023; Villena-Taranilla et al., 2022; Yu & Xu, 2022), as well as research conducted specifically on STEM fields (Liu et al., 2022; Martarelli, Dubach, et al., 2023). While the present study has focused on acceptance, it is important to highlight the need for further research on the effectiveness of IVR in primary education. Such research would not only advance our understanding of the potential benefits and drawbacks of IVR but would also further illuminate the suitability of IVR for young children. Importantly, bringing IVR into the classroom would facilitate the development of essential infrastructure and provide the necessary training for teachers. More importantly, it would allow stakeholders to gain firsthand experience with the technology, which we believe could positively influence attitudes toward the use of IVR in the classroom. We propose that implementation projects encouraging collaboration between researchers and schools could bridge the gap between IVR research and real-world school implementation. Additionally, involving parents/caregivers in this process may help to mitigate resistance and promote a more favourable view of IVR as a learning tool. Overall, to overcome existing scepticism toward IVR in primary education, it is essential to provide clear evidence of its benefits, along with robust support systems that empower directors and teachers.

This study has several limitations that should be acknowledged. First, we included only one to two items per UTAUT factor, which is fewer than those incorporated into many other studies, a trade-off we accepted to keep the questionnaire concise. In addition, the existing models (TAM and UTAUT), though widely used, were developed for contexts that differ from IVR implementation in primary schools. These models mainly focus on individual decision-making (whether or not to use a technology), and the wider context of schools, as well as the different stakeholders involved, are not taken into account. Future research should develop and empirically test updated models specific to this context. Second, the sample size, particularly for school directors (*n* = 37), was small; thus, the generalisability of the findings is limited. Third, the notable difference in sample sizes among the three groups hinders the ability to make inferential comparisons, as unequal group sizes can result in biased estimates of differences. Fourth, the study was conducted in a Swiss context, which may limit the applicability of the results to other educational systems or cultural contexts in which attitudes toward technology and digital media integration might differ. Finally, the use of a convenience sample means that the participants may not be representative of the wider population of school stakeholders. As a result, the findings should be interpreted with caution and future research with larger and more representative samples is needed to validate these results.

To conclude, digital media, such as computers and tablets, have become an integral part of everyday life, including in educational settings. Video games and other virtual environments have also been increasingly incorporated into learning, yet IVR remains rare in primary schools. While digital media offers educational benefits, it also comes with challenges. Successful integration of such technologies requires not only the appropriate technical infrastructure but also adequate teacher training. In this paper, we have emphasised the need for more research focused on the practical implementation of IVR in primary schools. Such research is essential for understanding the true benefits of IVR in educational contexts. Encouraging closer communication and collaboration between the scientific community and educational stakeholders is crucial to bridge this gap and to ensure that research not only informs but also facilitates the practical adoption of IVR in primary schools.

## Supplementary information

Supplementary Material

## Data Availability

The questionnaires, data, and code used in this study were made publicly available and can be accessed at the Open ScienceFramework at https://osf.io/jpq9n/.
