# Supplementary material for: Are primary schools ready for immersive virtual reality? Resistance among stakeholders
Source: Humanit Soc Sci Commun. 2025 Aug 12;12(1):1304. doi: 10.1057/s41599-025-05702-1 (PMC12343294; doi:10.1057/s41599-025-05702-1)
Supplement: Supplementary file 1 — Supplementary Material [file 41599_2025_5702_MOESM1_ESM.docx]

**Supplementary Material**

**Sample description**

*Directors*. We had 37 directors (11.97%, 18 women, mean age = 47.76 (*SD* = 13.18)) participate in our study. On average, the schools led by the directors had 15.73 (*SD* = 10.64) classes, 37.43 (*SD* = 25.52) teachers, and 312.65 (*SD* = 224.44) students. Directors had an average of 8.95 (*SD* = 7.40) years of professional experience in school management. Among the directors, 67.57% had never used IVR, 29.73% used it once a month or less, no one used it once or several times a week, and 2.70% used it daily. Of those who had used IVR at least once, no one found the experience very negative, 4.76% found the experience negative, 42.86% found it neutral, 38.10% found it positive, and 14.29% found it very positive.

*Teachers*. 70 teachers (22.65%, 48 women, mean age = 40.11 (*SD* = 14.66)) took part in our study. Among them, 67.14% taught German, 31.43% French, 31.43% English, 77.14% mathematics, 74.29% nature/people/society, 50.00% visual arts, 40.00% textile arts, 45.71% music, 62.86% physical education, and 11.43% other subjects. A total of 34.29% taught the first grade, 45.71% taught the second grade, 42.86% taught the third grade, 47.14% taught the fourth grade, 25.71% taught the fifth grade, and 30.00% taught the sixth grade. On average, the teachers had 14.41 (*SD* = 11.01) years of teaching experience. Of the teachers included, 82.86% used tablets in the classroom, 68.57% used laptops, 32.86% used desktop computers, 41.43% used smartphones, 22.86% used TVs, 1.43% used IVR headsets, 20.00% used other media, and 4.29% used no media. Privately, 61.43% of teachers used tablets, 92.86% used laptops, 38.57% used computers, 92.86% used smartphones, 71.43% used TVs, 7.14% used IVR headsets, 8.57% used other media, and no one used no media. Of the teachers included, 81.43% had never used IVR, 14.29% used it once a month or less, 2.86% used it once a week, 1.43% used it several times a week, and no one used it daily. Of those who had used IVR at least once, 4.55% had a very negative experience, 9.09% had a negative experience, 22.73% had a neutral experience, 36.36% had a positive experience, and 27.27% had a very positive experience.

*Parents/caregivers.* A total of 202 parents/caregivers (65.37%, 136 women, mean age = 44.06, *SD* = 11.37) were included in the study. The average age of their children was 9.24 (*SD* = 3.65) years. Of the children, 75.25% used tablets, 44.06% used laptops, 20.79% used computers, 55.45% used smartphones, 73.27% used TVs, 2.97% used IVR headsets, 12.38% used other media, and 2.97% used no media. Of the parents/caregivers, 58.42% used tablets, 83.17% used laptops, 49.01% used computers, 94.06% used smartphones, 76.24% used TVs, 5.94% used IVR headsets, 3.47% used other media, and 0.50% used no media. Again, of the parents/caregivers, 66.34% had never used IVR, 25.25% used it once a month or less, 3.96% used it once a week, 1.98% used it several times a week, and 2.48% used it daily. Of those who had used IVR at least once, 5.00% had a very negative experience, 10.00% had a negative experience, 38.33% had a neutral experience, 37.50% had a positive experience, and 9.17% had a very positive experience.

**One-sample t-tests**

In the main text, we focused on the distribution of the answers. When considering the means and the results of the one-sample t-tests against the chance level (50), we obtained similar outcomes. We report these results in the following table.

| **Item** | **Stakeholder** | ***M*** | ***SD*** | ***t*** | ***df*** | ***p*** | **Cohen’s d** |
| --- | --- | --- | --- | --- | --- | --- | --- |
| Virtual reality (VR) applications should be integrated into primary school teaching. | Directors | 47.32 | 28.66 | -0.57 | 36 | .574 | 0.09 |
|  | Teachers | 46.50 | 30.30 | -0.97 | 69 | .337 | 0.12 |
|  | Parents/caregivers | **36.54** | **34.46** | **-5.55** | **201** | **< .001***** | **0.39** |
| VR has great potential to improve learning in primary school. | Directors | 47.65 | 25.56 | -0.56 | 36 | .579 | 0.09 |
|  | Teachers | 51.74 | 28.54 | 0.51 | 69 | .611 | 0.06 |
|  | Parents/caregivers | **38.83** | **32.43** | **-4.89** | **201** | **< .001***** | **0.34** |
| The use of VR in teaching carries risks. | Directors | **65.19** | **23.48** | **3.94** | **36** | **< .001***** | **0.65** |
|  | Teachers | **65.40** | **30.98** | **4.16** | **69** | **< .001***** | **0.50** |
|  | Parents/caregivers | **65.70** | **30.28** | **7.37** | **201** | **< .001***** | **0.52** |
| I am confident that VR can be practically applied in primary school. | Directors | 54.11 | 29.99 | 0.83 | 36 | .410 | 0.14 |
|  | Teachers | 51.84 | 30.02 | 0.51 | 69 | .609 | 0.06 |
| I have the necessary resources to be able to use VR at my school. | Directors | **24.19** | **25.98** | **-6.04** | **36** | **< .001***** | **0.99** |
|  | Teachers | **20.39** | **27.10** | **-9.14** | **69** | **< .001***** | **1.09** |
| I have the necessary knowledge to be able to use VR at my school. | Directors | **25.68** | **31.03** | **-4.77** | **36** | **< .001***** | **0.78** |
|  | Teachers | **23.63** | **28.96** | **-7.62** | **69** | **< .001***** | **0.91** |
| In order to use VR applications in teaching, teachers need further training/introduction. | Directors | **90.05** | **19.17** | **12.71** | **36** | **< .001***** | **2.09** |
|  | Teachers | **87.33** | **19.21** | **16.26** | **69** | **< .001***** | **1.94** |
| I think it is easy to use VR in teaching. | Directors | 45.76 | 27.26 | -0.95 | 36 | .350 | 0.16 |
|  | Teachers | **37.29** | **25.12** | **-4.24** | **69** | **< .001***** | **0.51** |
| People whose opinions are important to me support the use of VR at my school. | Directors | **37.95** | **31.94** | **-2.30** | **36** | **.028*** | **0.38** |
|  | Teachers | **36.06** | **25.27** | **-4.62** | **69** | **< .001***** | **0.55** |
| How likely is it that you will use VR applications in your school in the future? | Directors | **41.68** | **24.89** | **-2.03** | **36** | **.049*** | **0.33** |
|  | Teachers | 44.04 | 29.48 | -1.69 | 69 | .095 | 0.20 |
| How strongly do you support the use of VR in primary school classes for your child/children? | Parents/caregivers | **37.58** | **33.95** | **-5.20** | **201** | **< .001***** | **0.37** |

**Table 1. Descriptive statistics and one-sample t-test results for different items, separated by directors (n = 37), teachers (n = 70), and parents/caregivers (n = 202).** For each item, we report the mean (*M*), standard deviation (*SD*), *t*-value, degrees of freedom (*df*), *p*-value, and Cohen’s d. The items are presented in the same order as in Figure 1. Note that while the questionnaire used the term “VR”, it was explicitly defined as IVR (VR presented through headsets) to ensure participant understanding. The t-tests were conducted against the chance level (50), with significant results highlighted in bold. **p* < .05. ****p* < .001.
